# Emotional Burnout, Perceived Sources of Job Stress, Professional Fulfillment, and Engagement among Medical Residents in Malaysia

**DOI:** 10.1155/2013/137620

**Published:** 2013-11-07

**Authors:** Sami Abdo Radman Al-Dubai, Kurubaran Ganasegeran, Wilson Perianayagam, Krishna Gopal Rampal

**Affiliations:** ^1^Department of Community Medicine, International Medical University (IMU), No. 126, Jalan Jalil Perkasa 19, Bukit Jalil, 57000 Kuala Lumpur, Malaysia; ^2^International Medical School, Management and Science University (MSU), University Drive, Off Persiaran Olahraga, Section 13, Shah Alam, 40100 Selangor, Malaysia; ^3^Medical Department, Tengku Ampuan Rahimah Hospital (HTAR), Jalan Langat, Klang, 41200 Selangor, Malaysia; ^4^Perdana University Graduate School of Medicine, Perdana University, Maeps Building, Mardi Complex, Serdang, 43400 Selangor, Malaysia

## Abstract

This study was the first to explore factors associated with emotional burnout (EB) among medical residents in Malaysia. A cross-sectional study was conducted in a universal sample of 205 medical residents in a Malaysian general hospital. The self-administered questionnaire used consisted of questions on sociodemographics and work characteristics, sources of job stress, professional fulfillment, engagement, and EB. EB was measured using the emotional exhaustion subscale, the Maslach Burnout Inventory (MBI). Mean (±SD) age of the respondents was 26.5 (±1.6). The most common source of job stress was “fear of making mistakes.” Most of the participants were dissatisfied with the increase of residentship period from one year to two years. A high level of EB was reported by 36.6% of the respondents. In multivariate analysis, the most important correlates of EB were sources of job stress, professional fulfillment, and engagement. A high prevalence of EB was found among medical residents. Sociodemographic characteristics, performance pressure, and satisfaction with policies were significantly associated with EB. Although this study was limited by its cross-sectional design, its findings posit a sufficient foundation to relevant authorities to construct, amend, and amalgamate existing and future policies.

*Nothing will sustain you more potently than the power to recognize in your humdrum routine, as perhaps it may be thought, the true poetry of life—the poetry of the common place, of the common man, of the plain, toil-worn woman, with their loves and their joys, their sorrows and their grief.*

SirWilliam Osler, Aphorisms from the Student Life (Aequanimitas, 1952)

## 1. Introduction

The modern medical workplace is a complex environment with intense drama [1]. Routine work strain with influx of patient admissions, responsibility of critical decisions, potential serious consequences, and pressure to avoid medical errors were among different facets of job conditions that have rendered medical practice inherently stressful [2].

The evolution from clinician centered to patient centered care in the modern medical workplace has challenged medical professionals to uphold integrity in providing quality health care and patient satisfaction [3]. Pressure on medical professionals has risen because of different health care reforms affecting clinicians' autonomy, prestige, personality, and income resulting in higher work stress and burnout [3].

Medical residency is a period of apprenticeship that transforms an academically qualified medical student into a competent medical practitioner. It prepares a newly graduated medical practitioner to be fully conversant and confident with the daily routines, workload, and pressures expected to be faced during clinical practice [4]. Such training, under close supervision by a senior attending physician, demands increased expectations and responsibilities [5]. Being first line service providers in a health facility, medical residents are expected to be proficient clinicians, educators, researchers, and administrators at the end of their residency training [6].

The intense emotional, psychological, and physical demands during residency were aimed to nurture caring, dynamic, and competent medical professionals towards enhancing patient centered care. Despite such noble intentions and goals, these efforts have led to opposite consequences and effects. The majority of clinicians from both developed and developing countries quit from medical career due to over stress [7]. Medical residents were overburdened with work expectations causing negative health effects [8]. It dampens motivation causing reduced concentration, attention, and impaired cognitive function [9]. These effects predispose residents to medical errors and injuries [8], substance abuse [10], conflict among colleagues [11], and suicide attempts [12].

Emotional burnout includes feelings of being overextended and depleted of one's emotional resources due to exhaustion from one's work [13]. Studies that explored physicians' emotional burnout identified various organizational outcomes leading to poor productivity and mood disturbances due to depleted resources [14, 15]. McManus et al. [16] found a reciprocal causation between emotional burnout and stress in a 3-year longitudinal study among UK clinicians. Previous cross-sectional study among general practitioners concluded that work satisfaction, social and economic prestige, and professional relations were associated with mental health well-being of the clinicians [17].

There is a lack of studies on burnout among medical residents in Malaysia. The Director General of Health Malaysia reported that 20% of medical residents in 2008 suffered from mental illness during residency training [18] and this figure increased to 31% in 2011 [4].

Yusoff et al. [4] found that high stress among medical residents in Malaysia was related to performance pressure. Aminah [19] concluded that residents' work hours and duties should be re-evaluated to prevent personal or family conflicts and preserve their health well-being. Medical residents are junior practitioners undergoing internship training under the Medical Act 1971. They are individuals who possess a recognized medical qualification, being provisionally registered with the Malaysian Medical Council (MMC) for two years to undertake four monthly postings in medicine, pediatrics, surgery, orthopedics, obstetrics and gynecology, and emergency medicine at approved public health facilities within Malaysia. The Graduate Medical Officer Flexi Timetable Work System Policy recently announced by the Malaysian government was aimed to improve medical residents' training quality through implementation of shift work system and to provide sufficient relaxation time [20]. This study was the first in Malaysia that aimed to explore factors associated with emotional burnout among medical residents in Malaysia, with particular focus on sources of job stress and professional fulfillment and engagement.

## 2. Materials and Methods

### 2.1. Study Setting and Population

This cross-sectional study was conducted among 205 medical residents at the Tengku Ampuan Rahimah Hospital (HTAR) Klang, the country's second busiest public health facility in terms of patient admissions [21]. All medical residents in the hospital at the time of the study were approached by using a universal sampling technique. After arrangement with relevant head of departments and hospital management, residents from all six major departments (Medicine, Obstetrics & Gynecology, Surgery, Emergency Medicine, Pediatrics, and Orthopedics) were approached through the chief resident during hospital Continuous Medical Education (CME) sessions and after working hours. Objectives and benefits of the study were explained verbally to the chief resident and in a written form attached to the questionnaires. Respondents were assured that information obtained would be confidential and their participation would not affect their progress during residency. A written consent was obtained from those who agreed to participate. To ensure that only medical residents participated in this study, we requested the provisional license number of the residency to be indicated in the consent form. This number was entered into the Malaysian Medical Council (MMC) provisional registration database to ensure respondents validity.

### 2.2. Ethical Issues

This research protocol was approved by the Ethics Committee of the National Institutes of Health, Ministry of Health Malaysia (government approval number NMRR-11-1128-9716).

### 2.3. Study Instruments

The self-administered questionnaire consisting of four parts was used in this study. 

#### 2.3.1. Sociodemographics and Work Characteristics

This part included questions on gender, age, ethnicity, marital status, smoking and alcohol, university graduated, graduate qualification, current duration of residency training, current rotation, and working hours with the government per week. 

#### 2.3.2. Sources of Job Stress

Sources of job stress were assessed by eighteen validated items adapted from previous published studies [7, 14, 22, 23]. Participants were asked “Do you think the following aspects causes stress to you?” Response for each item was optioned “Yes” or “No.” It included statements like “fear of making mistakes,” “work overload and on-call,” “worries about finances,” and “feeling insecure in this job.”

#### 2.3.3. Professional Fulfillment and Engagement

This study defined professional fulfillment as career contentment that one seeks reward to elucidate a sense of engagement [24]. Engagement was defined as the orthogonally arranged positive and negative conditions of burnout, making medical practice less of a job and more of a passion with engaged employees being energetic and enthusiastically applying their energy to work [25] through work satisfaction policies. Professional fulfillment and engagement was assessed through satisfaction with salary or extra incentives, increase of residency period from 1 year to 2 years, learning experience, the 2-way-feedback/report resident-supervisor system, and the Graduate Medical Officer Flexi Timetable Work System Policy by the Malaysian government (Prior Flexi-Work Hours Policy, per on-call was paid MYR 100.00. Following Flexi-Work Hours Policy, on-call was substituted with shift work and Flexi-Work-Hours Allowance was introduced with a flat rate of MYR 600.00 per month.). This policy initiated the introduction of the “Flexi-Work-Hours Policy” in Malaysia, “substitution of on-call with shift work,” “decrease working hours to 60–72 hours per week,” introduction of “two days off per week,” and “replacement of on-call allowance with Flexi-Work-Hours Allowance” [21]. Participants were asked if they were satisfied with the previous items with a response option of “Yes” or “No.” 

#### 2.3.4. Emotional Burnout

To assess emotional burnout, we used the Emotional Exhaustion (EE) subscale of the validated Maslach Burnout Inventory (MBI) [1], a widely used global assessment tool for burnout. Emotional Exhaustion (EE) subscale (the feelings of being emotionally over-run and exhausted by one's work) is the most significant dimension of burnout in the MBI [26]. The 9 items of EE were answered in terms of frequency in which the respondent experiences these feelings, on a 7-point Likert scale ranging from 0 (never) to 6 (every day). A higher score indicated greater emotional exhaustion [27] and, accordingly, a higher emotional burnout. The Cronbach's alpha coefficient of the emotional exhaustion subscale reported in the literature was satisfying (0.88) [26]. In this study, Cronbach's alpha coefficient of this scale was 0.90. The exploratory factor analyses yielded one factor with given value greater than 1 (5.0). The one-factor solution accounted for 55.5% of the variance.

### 2.4. Statistical Methods

The Statistical Package for Social Sciences (SPSS) version 16.0 was used to analyze data in this study. Descriptive analysis for sociodemographics, perceived sources of job stress, and professional engagement was performed. The 9 items of emotional exhaustion subscale were summed to obtain the total score (0 to 54). A high degree emotional burnout was based on the cut-off point of the emotional exhaustion subscale in the MBI (≥27) [14]. Test of normality of the total score of emotional exhaustion was conducted. Student's *t*-test and ANOVA were used to compare the mean of emotional burnout score across demographic variables, perceived sources of job stress, and items of professional engagement. Multivariate linear regression using “Backward” technique was employed to obtain factors associated significantly with emotional burnout score. Variables that were significantly associated with burnout in the bivariate analysis were included in the multivariate analysis. Multicollinearity between independent variables was checked for by the values of standard errors (SE) not exceeding 5. The accepted level of significance was set below 0.05 (*P* < 0.05).

## 3. Results

### 3.1. Sociodemographic Characteristics

One hundred and ninety-one out of 205 medical residents gave consent to participate in this study with a response rate of 93.2%. The fourteen nonrespondent residents composed of eight females and six males. Majority of the respondents were females (55.5%) and aged between 25 and 27 years old (65.4%). Most of them were Chinese (35.6%), while Malays and Indians constituted 34.0% and 30.4%, respectively. The majority were single (80.6%), graduated medical degree with a “pass” (71.7%), and were from local public institutions of higher learning (41.4%). More than half (59.7%) were rotating in their second year of medical residency. Majority of them worked for 60 hours over the past week (61.3%) (Table 1).

### 3.2. Perceived Sources of Job Stress among Respondents

The five most important stressors reported by majority of the respondents were “fear of making mistakes” (90.6%), “time pressures and difficulty to meet deadlines” (74.9%), “working with uncooperative and incompetent colleagues” (74.3%), “lack of adequate comfortable rest rooms and other facilities for doctors” (73.8%), and “lack of incentives and promotions” (73.8%).

### 3.3. Professional Fulfillment and Engagement among Respondents

The majority were satisfied with the following efforts to be professionally engaged: introduction of a two-way-feedback/report system of resident supervisor relationship (91.1%), decrease of working hours to 60–72 hours with a “two-days-off” per week (90.1%), salary rise (89.0%), introduction of the “Flexi-Work-Hours Policy” in Malaysia (84.8%), and the substitution of “on-call” with “shift work” (83.2%). The majority of residents (73.8%) were satisfied with the overall learning experience during medical residency period. More than half (54.5) were dissatisfied with the increase of residentship period from one year to two years (Table 2).

### 3.4. Emotional Burnout

Mean (±SD) of emotional burnout score was 23.1 (±10.4) and the score ranged from 0 to 54. Seventy residents (36.6%) experienced high level of emotional burnout.

### 3.5. Association between Sociodemographic Factors and Emotional Burnout

Mean (±SD) total score of emotional burnout was compared across different categorical variables. There was a significant association between posting rotations and emotional burnout among medical residents (*P* = 0.008); post hoc test revealed that those rotating in Obstetrics & Gynecology department (30.2 ± 12.8) had higher emotional burnout compared to Medical (22.8 ± 7.3), Orthopedics (21.5 ± 11.2), and Surgical (19.5 ± 11.7) departments (*P* = 0.045, *P* = 0.003, and *P* = 0.038, resp.) (Table 3). 

### 3.6. Association between Perceived Sources of Job Stress and Emotional Burnout

Mean and (±SD) total emotional burnout score was compared between those who answered “yes” and those who answered “no” on each source of stress.  Table 4 showed that sixteen out of eighteen sources of job stress exhibited significant association with emotional burnout (*P* < 0.05).

### 3.7. Association between Professional Fulfillment and Engagement and Emotional Burnout

Mean and (±SD) total emotional burnout score was compared between those being “satisfied” and “unsatisfied” on each item of professional fulfillment and engagement. Four out of nine items were significantly associated with emotional burnout (*P* < 0.05) (Table 5). 

### 3.8. Factors Associated with Emotional Burnout among Medical Residents in Multiple Linear Regression Analysis

Medical residents who graduated medical school with a “pass” had on the average 2.8 (95% CI 0.1–5.5) higher score in emotional burnout compared to those graduated with a “distinction” (*P* = 0.045). Malays had on the average 3.7 (95% CI 0.7–6.8) higher score in emotional burnout compared to Indians (*P* = 0.017). Age was significantly associated with emotional burnout (*P* = 0.041). Medical residents who cited work demands as affecting their personal or home life had on the average 3.5 (95% CI 0.6–6.4) higher score in emotional burnout compared to medical residents who denied such claims (*P* = 0.019). Medical residents who had difficulty maintaining good relationships with their supervisor had on the average 4.3 (95% CI 1.5–7.1) higher score in emotional burnout compared to those maintaining good resident-supervisor relationships (*P* = 0.003). Medical residents who felt underpaid had on the average 5.3 (95% CI 2.4–8.1) higher score in emotional burnout compared to those being satisfied with their wages (*P* < 0.001). Medical residents who were satisfied with the reduction of work duration to 60–72 hours and a “two days off” per week had on the average 6.5 (95% CI 2.4–1.0) lower score in emotional burnout compared to those who were unsatisfied with such policy (*P* = 0.002). Those being in favor with the introduction of resident-supervisor feedback system had on the average 9.7 (95% CI 13.9–5.4) lower scores in emotional burnout compared to residents' who disapproved such implementation (*P* < 0.001) (Table 6). Analysis showed no intercorrelation between independent variables. The total model was significant (*P* < 0.001) and the adjusted *R* square was 0.35 which means that the variables in this model explained 35% of the variance on the emotional burnout among medical residents.

## 4. Discussion

This study considered the mounting evidence of medical residents emotional burnout that adversely affect residents' mental health well-being. The government of Malaysia had indeed embarked on an aggressive effort to improve medical residents' situation, for example, the implementation of Graduate Medical Officer Flexi Timetable Work System Policy. This study has attempted to explore the level of burnout and its association with sources of job stress and professional engagement. 

Data on prevalence rates of emotional exhaustion among medical residents in Asian countries is scarce. This study is the first to estimate the prevalence and associated factors of emotional burnout among medical residents in Malaysia. This study found that 36.6% of residents had a high level of emotional burnout during residency training. Prevalence rates in western countries were found to be ranged between 13.0% and 56.6% [28, 29]. There is an increasing number of students graduating from medical schools in Malaysia as well as Malaysian students graduating from medical schools in other countries including India, Indonesia, Egypt, and Russia who are returning to Malaysia to practice. Except for a new Graduate Entry Medicine (GEM) Doctor of Medicine Program in Malaysia, whose first batch of students are expected to graduate in 2015, all of these medical residents would have undergone a five- or six-year medical degree program from or outside Malaysia. Those graduating from Malaysian medical schools and from the Malaysian Medical Council's list of recognized medical schools outside Malaysia can directly undergo the two-year house officer (medical resident) training described. Those from medical schools that are not recognised have to attach themselves to medical schools in Malaysia specified by the Malaysian Medical Council and sit for the final year examination of these schools. Currently, there is a global trend towards Graduate Entry Medicine with medical schools in the United Kingdom, Australia, Ireland, and Singapore conducting GEM programs solely or in parallel with schools leaver programs. 

Reports on the association between sociodemographic characteristics and emotional burnout among medical residents were subjective as the majority of the published work proposed weak or negative correlations [29, 30]. Our study findings were similar. With respect to working conditions, this study found a significant association between working hours and emotional burnout. This finding is similar to the results of a recent study among Turkish residents [31]. A significant association was seen between residents' rotations and emotional burnout. Shanafelt et al. [28] had similar findings.

A myriad of stress factors was significantly associated with emotional burnout among medical residents in this study. Consistent with previous literature, various factors like work overload [4], work environment and available resources [7, 21], and remunerations and incentives [17] showed positive relationships with residents' emotional burnout. A new observation found in this study was the significant association between resident-supervisor relationship and emotional burnout. This study postulates that appropriate mentorship, sufficient motivation, and fair assessments during resident-ship are key to preventing emotional burnout among medical residents. 

The scientific literature has shown that *t* burnout prompted serious personal repercussions like substance abuse [32], family conflicts [19], and suicidal ideation [28]; much catastrophically compromising the efficacy of healthcare delivery system through increased medical errors [33]. This study found emotional burnout to significantly associate with residents' personal and home life. Previous studies found similar findings [30, 34].

Another interesting new observation in this study is the association between residents' emotional burnout and professional engagement through domains of the newly Graduate Medical Officer Flexi Timetable Work System Policy in Malaysia. Medical residents' who were satisfied with the implementation of resident-supervisor-feedback/report system, reduction of work hours to 60–72 hours with a “two days off per week,” and the introduction of Flexi-Work-Hours Policy exhibited a significantly lower level of emotional burnout compared to those not satisfied. Those who were unsatisfied with the increase of residency period from 1 year to 2 years exhibited a significantly higher level of emotional burnout in comparison to satisfied ones. 

An important finding in this study is having vocation (engagement) and avocation (leisure) is beneficial to professional behavior. We found a significant association between emotional burnout and work duration with adequate rest days per week for residents' leisure activities. While this finding is consistent with those expressed by Osler [35] and justified to be essential by Ozyurt et al. [1] that avocation would benefit doctors by increasing their sense of vocation and preventing emotional burnout, McManus et al. [25] concluded otherwise.

Reward is an important factor influencing the motivation to work [36] and preventing mental well-being deterioration of work stress and emotional burnout [17]. The Malaysian Remuneration System (MRS) which replaced the New Remuneration System (NRS) scheme in 2002 showcased a revised adjustment of 13 percent salary increase from January 2012 for the medical and health professionals group (Grade UD41-54) [37]. Despite salary adjustments, this study found significant association between residents' remuneration and emotional exhaustion. Bovier et al. [17] found similar findings. The Effort-Reward Imbalance Model necessitates the balance between work efforts and rewards to be executed through triadic domains: salary, prestige, and job security [38].

 The debate of house officers having inadequate clinical exposure during residency training has never been resolved since the health care emphasizing patient centeredness ventured into Malaysia. This study found that 73.8% of residents were satisfied with the overall learning experience during their training. Our finding was inconsistent with a recent survey conducted by the Malaysian Medical Association (MMA) (2012) [39] that the newly introduced Flexi-Work-Hours policy in Malaysia failed to provide adequate clinical exposure during resident-ship training. While our study is in line with the concern expressed by MMA that “medicine is a science of uncertainty and an art of probability” [40] and requires extensive clinical exposure to formulate appropriate judgments and decisions [40], skeptics failed to understand the principle that “the value of experience is not in seeing much, but in seeing wise” [35]. This study postulates that duration and time of clinical exposure during residency is not a reliable tool to measure resident competency, as medicine is a continuous learning process which requires commitment. 

### 4.1. Study Limitations

The cross-sectional nature of this study created difficulties in ascertaining causal relationships between variables. Self-reported data collected at one point in time necessitated care in drawing conclusions of the effects of working conditions on emotional burnout or professional engagement. Data obtained in this study was from a single hospital in Malaysia; this may affect the generalizability of the results to all medical residents in Malaysia. A larger sample should be required in future studies to avoid possible selection bias. 

## 5. Conclusion

The most common sources of job stress among medical residents were fear of making mistakes, time pressures and difficulty in meeting deadlines, working with uncooperative and incompetent colleagues, and lack of adequate comfortable facilities for doctors. Most of the residents were satisfied with all aspects of the work policies and directives except “the increase of medical residency period from one year to two years.” About a third of the medical residents in this study had a high level of emotional burnout. Important factors associated with emotional burnout were age, residents graduated medical degree with a “pass,” being Malay, increasing work demands, feeling of being underpaid, and reciprocal resident-supervisor relationships. 

## 6. Recommendations

With the paradigm shift towards American style graduate entry medical programs that facilitates clinical exposure at early years in Malaysian medical schools, this study suggests to revert medical residency period to one year. This would enable residency training during undergraduate medical education rather than to impose it after graduation. The role of supervisors should be changed from “evaluators of medical resident competencies” to “mentors” that coach, advice, care, and counsel their medical residents through excellent supervisor-resident rapport. These changes are required to mould medical residents to be competent and adaptive in medical practice as well as to reduce their mental woes.

## Figures and Tables

**Table 1:** Sociodemographics of the respondents (*n* = 191).

Characteristics	*n* (%)
Gender	
Male	85 (44.5)
Female	106 (55.5)
Age	
≤24	17 (8.9)
25–27	125 (65.4)
≥28	49 (25.7)
	Mean = 26.5, SD = 1.6, Min = 23, Max = 33
Ethnicity	
Malay	65 (34.0)
Chinese	68 (35.6)
Indian	58 (30.4)
Marital status	
Single	154 (80.6)
Married/cohabiting	37 (19.4)
University graduated	
Local public	79 (41.4)
Local private	39 (20.4)
International	73 (38.2)
Graduate qualification	
Distinction	13 (6.8)
Honors	41 (21.5)
Pass	137 (71.7)
Current month of residency training	
≤12 months	73 (38.2)
13–24 months	114 (59.7)
>24 months	4 (2.1)
Current rotation	
Obstetrics & Gynecology	23 (12.0)
Medicine	62 (32.5)
Surgery	30 (15.7)
Emergency Medicine	23 (12.0)
Pediatrics	25 (13.1)
Orthopedics	28 (14.7)
Work hours of the past week	
60 hours	117 (61.3)
72 hours	74 (38.7)
Smoking	
Yes	19 (9.9)
No	172 (90.1)
Alcohol	
Yes	22 (11.5)
No	169 (88.5)

**Table 2:** Professional fulfillment and engagement among respondents (*n* = 191).

Statement	Satisfied *n* (%)
Introduction of a two-way-feedback/report-system of resident-supervisor relationship during residency training.	174 (91.1)
Decrease working hours to 60–72 hours and the introduction of “a two days off” per week.	172 (90.1)
Salary rise.	170 (89.0)
Introduction of the “Flexi-Work-Hours Policy” in Malaysia.	162 (84.8)
Substitution of “on-call” with “shift work.”	159 (83.2)
Replacement of “on-call allowance” with “Flexi-Work-Hours Allowance.”	154 (80.6)
Overall satisfaction with learning experience during resident-ship.	141 (73.8)
Attractive incentives (free food from cafeteria, allocation of parking lots, comfortable room facilities, and additional days of annual leave and leisure).	121 (63.4)
Increase of residentship period from one year to two years.	87 (45.5)

**Table 3:** Association between sociodemographic factors and emotional burnout (*n* = 191).

Variables	Mean (SD)	*P* value
Gender		
Male	23.6 (10.9)	0.529
Female	22.7 (10.0)	
Age		
≤24	21.8 (5.8)	0.850
25–27	23.3 (10.6)	
≥28	22.9 (11.1)	
Ethnicity		
Malay	24.4 (8.9)	0.077
Chinese	24.0 (10.4)	
Indian	20.5 (11.6)	
Marital status		
Single	22.5 (10.4)	0.132
Married/cohabiting	25.4 (10.0)	
University graduated		
Local public	23.1 (7.9)	0.614
Local private	21.8 (9.1)	
International	23.8 (13.1)	
Graduate qualification		
Distinction	23.1 (8.9)	0.060
Honors	19.7 (10.2)	
Pass	24.1 (10.4)	
Current month of residency training		
≤12 months	22.2 (9.5)	0.199
13–24 months	24.1 (11.5)	
>24 months	29.8 (9.6)	
Current rotation		
Obstetrics & Gynecology	30.2 (12.8)	0.008
Medicine	22.8 (7.3)	
Surgery	19.5 (11.7)	
Emergency Medicine	22.3 (9.9)	
Pediatrics	24.4 (10.2)	
Orthopedics	21.5 (11.2)	
Work hours over the past week		
60 hours	21.7 (10.2)	0.019
72 hours	25.3 (10.3)	
Smoking		
Yes	24.3 (10.9)	0.606
No	23.0 (10.4)	
Alcohol		
Yes	24.7 (11.9)	0.434
No	22.9 (10.2)	

**Table 4:** Association between emotional burnout and perceived sources of job stress among respondents (*n* = 191).

Sources of job stress	Emotional burnout mean (SD)		*P* value
	Yes	No	
Fear of making mistakes.	23.4 (10.5)	19.9 (8.5)	0.177
Time pressures and difficulty to meet deadlines.	25.0 (9.8)	17.6 (10.3)	<0.001
Working with uncooperative and incompetent colleagues.	24.2 (9.9)	20.0 (11.1)	0.016
Lack of adequate comfortable rest rooms and other facilities for resident doctors.	24.5 (9.7)	19.3 (11.4)	0.002
Lack of incentives and promotions.	24.9 (9.8)	18.1 (10.5)	<0.001
Feeling of underpaid.	25.1 (9.8)	17.8 (9.9)	<0.001
Feeling of inadequate knowledge and skills to meet work demand and objectives.	24.4 (9.8)	19.4 (11.1)	0.006
Work overload and “on-call.”	25.2 (9.6)	18.2 (10.7)	<0.001
Work demands affect personal and home life.	25.5 (9.8)	17.6 (9.6)	<0.001
Inadequate skills for dealing with more difficult aspects of work matters.	25.1 (10.0)	19.3 (10.1)	<0.001
Worries about finances.	24.3 (9.3)	21.7 (11.5)	0.084
Lack of support and unfair assessment from supervisor.	26.0 (9.7)	19.6 (10.1)	<0.001
Working outside one's competence.	26.2 (9.1)	19.7 (10.7)	<0.001
Fear of infection.	25.9 (9.8)	20.3 (10.3)	<0.001
Lack of resources.	25.2 (9.7)	21.0 (10.7)	0.005
Feeling unsafe during work.	26.3 (9.6)	20.4 (10.3)	<0.001
Feeling insecure in this job.	26.3 (8.7)	21.0 (10.9)	<0.001
Difficulty in maintaining relationship with supervisor.	27.5 (9.7)	20.3 (9.8)	<0.001

**Table 5:** Association between professional fulfillment and engagementand emotional burnout (*n* = 191).

	Emotional burnout mean (SD)		*P* value
	Satisfied	Unsatisfied	
Introduction of a two-way-feedback/report system of resident-supervisor relationship during residentship training.	22.3 (9.7)	30.8 (13.7)	0.001
Decrease of working hours to 60–72 hours and the introduction of “a two days off” per week.	17.8 (8.5)	23.7 (10.4)	0.020
Salary rise.	20.8 (10.9)	23.4 (10.3)	0.286
Introduction of the “Flexi-Work-Hours Policy” in Malaysia.	18.8 (9.2)	23.9 (10.4)	0.014
Substitution of “on-call” with “shift work.”	20.7 (10.7)	23.6 (10.3)	0.156
Replacement of “on-call allowance” with “Flexi-Work-Hours Allowance.”	20.5 (9.2)	23.7 (10.6)	0.089
Overall satisfaction with learning experience during medical residency.	22.7 (10.3)	24.2 (10.7)	0.374
Attractive incentives (free food from cafeteria, allocation of parking lots, comfortable room facilities, and additional days of annual leave and leisure).	22.2 (9.0)	24.6 (12.3)	0.121
Increase of residentship period from one year to two years.	21.2 (11.4)	25.4 (8.6)	0.005

**Table 6:** Factors associated with emotional burnout by multiple linear regression (*n* = 191).

	*B*	SE	Beta	*P* value	95% CI	
					Lower	Upper
Graduate qualification (pass)	2.8	1.4	0.1	0.045	0.1	5.5
Ethnicity (Malay)	3.7	1.6	0.2	0.017	0.7	6.8
Ethnicity (Chinese)	2.8	1.5	0.1	0.065	−0.2	5.8
Age	0.8	0.4	0.1	0.041	0.0	1.6
Work demands affect personal/home life.	3.5	1.5	0.2	0.019	0.6	6.4
Difficulty in maintaining relationship with supervisor.	4.3	1.4	0.2	0.003	1.5	7.1
Feeling of underpaid.	5.3	1.5	0.2	<0.001	2.4	8.1
Satisfaction with the decrease of working hours to 60–72 hours and a “two days off” per week.	−6.5	2.1	−0.2	0.002	−2.4	1.0
Satisfaction with the introduction of the two-way-feedback/report-system of resident-supervisor relationship during residency training.	−9.7	2.2	−0.3	<0.001	−13.9	−5.4

The reference group for graduate qualification is “Distinction”; for ethnicity is “Indian”; for satisfaction with the weekly work duration is “yes”; for all other variables is “no.”
